# Forensic Gender Prediction by Using Mandibular Morphometric Indices: A Panoramic Radiograph Study

**DOI:** 10.7759/cureus.56603

**Published:** 2024-03-20

**Authors:** Abirami Arthanari, Shanmathy Sureshbabu, Karthikeyan Ramalingam, Lavanya Prathap, Vignesh Ravindran

**Affiliations:** 1 Department of Forensic Odontology, Saveetha Dental College and Hospitals, Saveetha Institute of Medical and Technical Sciences, Saveetha University, Chennai, IND; 2 Department of Oral and Maxillofacial Pathology, Saveetha Dental College and Hospitals, Saveetha Institute of Medical and Technical Sciences, Saveetha University, Chennai, IND; 3 Department of Anatomy, Saveetha Medical College and Hospitals, Saveetha Institute of Medical and Technical Sciences, Saveetha University, Chennai, IND; 4 Department of Pediatric Dentistry, Saveetha Dental College and Hospitals, Saveetha Institute of Medical and Technical Sciences, Saveetha University, Chennai, IND

**Keywords:** mandibular metrics, sex determination, antegonial angle, gonial angle, mandibular parameters

## Abstract

Aim and objective

This study aims to assess the accuracy of gender estimation using gonial and antegonial angles and determine the gender of the given samples using gonial and antegonial angles.

Introduction

An essential component of the human skull, the mandible, exhibits sexual dimorphism, making it a valuable tool in forensic and anthropological investigations for detecting sex. This procedure can be especially difficult in situations where there are large numbers of casualties, natural disasters, or widely dispersed remains. The mandible is an accurate indicator of age and sex because it responds to patterns of growth. Sex determination through the gonial angle and antegonial angle involves analyzing the angles formed by the lower jaw bone in individuals. Examining these angles contributed to remarkable accuracy.

Materials and methods

A total of 500 samples, 250 male and 250 female panoramic radiographs, were carefully chosen for the study. The chosen samples ranged in age from 20 to 30 years. Orthophantomograms were measured in Planmeca software (Planmeca Romexis®, Version 6.0, USA INC.) and the descriptive statistics (paired t-test) were performed in SPSS Statistics version 16.0 (SPSS Inc. Released 2007. SPSS for Windows, Version 16.0. Chicago, SPSS Inc.).

Results

Gonial angle for males obtained the highest value of 127.27±5.29, followed by females at 127.08±5.18. This was followed by the antegonial angle for males of 10.74±3.14, and the least value was obtained by females of 10.67±3.30. The p-value for the gonial angle showed no statistical significance for males or females (p=0.679). Antegonial angle showed statistical significance in both males (p=0.008) and females (p=0.001).

Conclusion

Among both the parameters considered, the antegonial angle showed significance in determining the gender of the given samples. Hence, to assess the accuracy of gender estimation, the antegonial angle can be used as a parameter.

## Introduction

A critical component of bioarchaeology, archaeology, and forensic anthropology is estimating gender from skeletal remains. In situations where alternative kinds of identification are not accessible, it acts as a first step in the identification process [1]. The mandible is particularly important among the skeletal remains used to estimate gender because of its sexually dimorphic traits. Particularly, the gonial and antegonial angles of the mandible have drawn interest as trustworthy markers of sexual dimorphism, providing important information for identifying biological sex [2]. It is thought that sexual dimorphism is caused by variations in biomechanical loading, hormonal effects, and genetic factors between males and females. The gonial angle is formed at the intersection of the ramus and the mandibular body, while the antegonial angle is formed anteriorly from the intersection of the ramus and the mandibular body. These angles stand for angular measurements that can be measured with a variety of imaging techniques, such as cephalometric radiography, computed tomography (CT) scans, and cone-beam computed tomography (CBCT). Traditional anthropometric methods can also be used to quantify these angles [3].

Usually wider in males than in females, the gonial angle is defined as the angle formed by the ramus and the mandibular body. Males often have more prominent mandibular muscle attachments, which results in a stronger mandibular angle. This sexual dimorphism is related to differences in muscle origin and insertion [4]. On the other hand, because of their larger mandibles overall and comparatively smaller muscle attachments, females typically display a more obtuse gonial angle. Similarly, it has been discovered that the antegonial angle, which represents the inclination of the front mandibular body, is sexually dimorphic. Research has demonstrated that whereas females generally display a more obtuse antegonial angle, indicating a more horizontally oriented mandibular body, males generally have a more acute antegonial angle. Hormonal factors, especially testosterone, are thought to have an impact on these disparities because of their substantial role in the growth and development of the mandible throughout puberty [5].

There are various benefits to using gonial and antegonial angles in gender assessment. First of all, these measurements are very useful in forensic contexts where destructive sampling is not desired because they can be taken non-invasively from skeletal remains [6]. Second, a number of population-based studies have proven the accuracy and dependability of these angles, confirming their applicability to a wide range of demographic groupings. Further, the accessibility of digital imaging technologies and the ease of measuring have made it easier for this technology to be widely used in forensic contexts. Investigations are still being conducted to improve and confirm methods for estimating gender from mandibular angles [7]. Using sophisticated imaging techniques like geometric morphometrics and three-dimensional surface scanning can improve the precision and accuracy of angle measurements. Moreover, multidisciplinary cooperation among radiologists, computer scientists, and anthropologists has enabled the creation of automated algorithms for angle quantification, simplifying the analysis procedure and lowering subjectivity [8]. The aim and objective is to assess the accuracy of gender estimation using gonial and antegonial angles. To determine the gender of the given samples, use the gonial angle and the antegonial angle.

## Materials and methods

**Figure 1:**
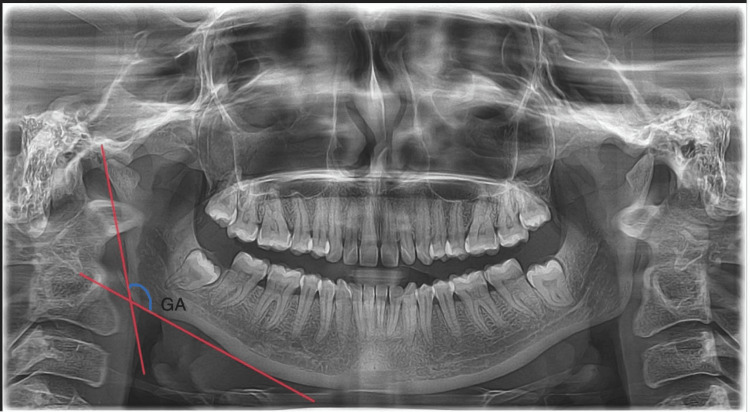
Given radiograph represents the gonial angle formed between the posterior border and inferior border of the mandible

The present study was carried out in the Department of Forensic Odontology at Saveetha Dental College and Hospital. Samples were collected from the archives of the Department of Oral Medicine and Radiology at Saveetha Dental College and Hospital. A randomly selected 500 samples (250 males and 250 females) were collected, aged between 20 and 30 years, using a standard digital orthopantomogram. Cases with known sex and the availability of orthopantomogram radiographs for each sample with head alignment that was contrasting and clearly visible were included in the study. Radiographs with artifacts and pathological conditions were excluded from the study. The parameters used here were measured by using Planmeca software (Planmeca Romexis®, Version 6.0, USA INC.). With the use of SPSS Statistics version 16.0 (SPSS Inc. Released 2007. SPSS for Windows, Version 16.0. Chicago, SPSS Inc.), statistical analysis for the data was analyzed. The Institutional Human Ethics Committee of Saveetha Dental College approved this study (approval number: IHEC/SDC/FACULTY/22/FO/059). Using G-Power software (version 3.1.9.4, Düsseldorf, Germany), the sample size was determined to guarantee a 95% statistical power and a 0.05 significance threshold. A total of 500 samples were included, with a calculated sample size of 482. The gonal angle was measured in the present study as represented in Figure 1.

**Figure 2:**
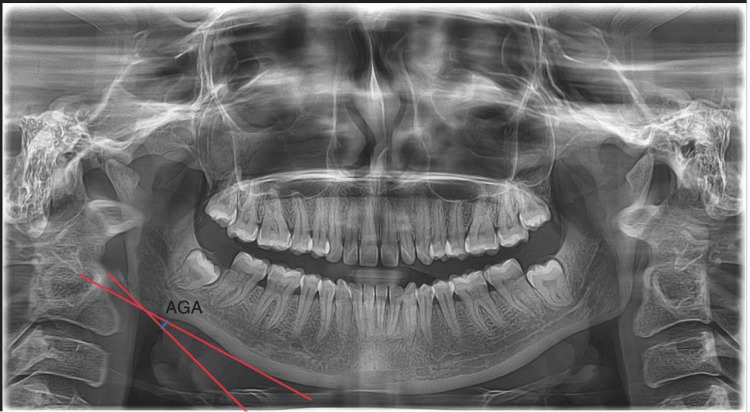
Given radiograph represents the antegonial angle formed between the posterior border and inferior border of the mandible

The antegonial angle was measured in the present study, as represented in Figure 2.

## Results

The mean comparison between males and females based on the gonial angles revealed males exhibited the highest mean value (127.27 ± 5.29) and females had a slightly lower mean value (127.08 ± 5.18). The p-value for the gonial angle indicated no statistical significance between males and females (p=0.679). Antegonial angle in males had a higher mean value (10.74 ± 3.14). Females showed a lower mean value (10.67 ± 3.30). The mean comparison between males and females based on both males and females demonstrated statistical significance in the antegonial angle, with p-values of 0.008 and 0.001, respectively. Overall, while there was no statistically significant difference between males and females in terms of the gonial angle, there was a significant difference in the antegonial angle, as given in Table 1.

**Table 1:** Mean comparison between males and females according to the gonial angle and antegonial angle

Group statistics						p-value
Gender		N	Mean	Std. Deviation	Std. Error Mean	
Gonial angle (degree)	Female	250	127.075	5.17621	0.32737	0.679
	Male	250	127.2685	5.28657	0.33435	0.679
Antegonial angle (degree)	Female	250	10.6651	3.30289	0.20889	0.001
	Male	250	10.7387	3.13757	0.19844	0.008

**Figure 3:**
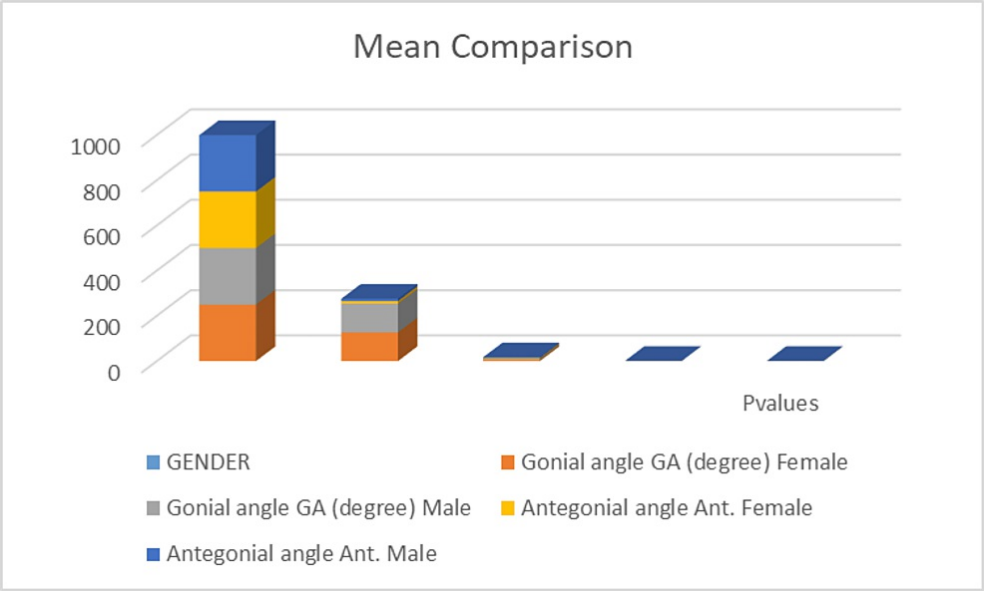
Mean comparison between males and females according to gonial angle and antegonial angle

The results show mean comparison values between males and females according to the gonial angle and antegonial angle (in degrees). These results indicate that while there was no significant difference between males and females in terms of gonial angle, there was a statistically significant difference in the antegonial angle represented in Figure 3.

According to the confidence intervals of gonial angle in males and females, they varied, ranging from -1.11 to 0.73 for both males and females, which is represented in Table 2.

**Table 2:** Confidence interval for gonial angle between males and females

95% Confidence Interval of the Difference			
Mandible parameter	Gender	Lower	Upper
Gonial angle	Female	-1.11289	0.72585
	Male	-1.11289	0.72585

The canonical discriminant function showed a statistical significance of 0.000 in Table 3.

**Table 3:** Summary of canonical discriminant function

Test of Function(s)	Wilk’s Lamba	Chi-square	df	Sig.
1	0.502	340.407	9	0.000

The accuracy of the gonial angle is about 0.090, and the antegonial angle is about -0.114, as represented in Table 4.

**Table 4:** Standardized canonical discriminant function coefficient By using the gonial angle, an Indian-specific regression formula was derived from our study: gender = 0.090*gonial angle, -0.114*antegonial angle

Mandible Parameters in Degree	Function (1)
Gonial angle (degree)	0.090
Antegonial angle (degree)	-0.114

## Discussion

A key component of forensic anthropology is gender estimation using human skeletal remains, which depends on a number of anatomical traits that display sexual dimorphism. Gender cannot be determined with certainty from a single skeleton element, but a combination of traits yields a more accurate estimate [9]. First, the morphology of the mandible varies greatly both within and between individuals. The heterogeneity of this data can be attributed to various factors, including age, ancestry, and environmental influences. Therefore, it is difficult to determine universal cutoff values for sex identification strictly based on these angles. The larger sciatic notch, subpubic angle, and pelvic inlet shape are some of the skeletal characteristics that are thought to be the most sexually dimorphic in the pelvis. These characteristics tend to be narrower and more closed in men but wider and more open in females [10]. The skull also provides useful hints for estimating gender. Male craniums are usually more sturdy, with larger mastoid processes and more noticeable muscle attachments. Men typically have a more prominent brow ridge, while women typically have a smoother forehead. The size and form of the mandible are other characteristics of the cranium that are sexually dimorphic, with males usually having larger mandibles than females [11]. The humerus and femur are two examples of long bones that exhibit sexual dimorphism. Because they normally have more muscular mass and strength, men tend to develop longer, thicker bones with larger muscle attachment sites. Gender assessment can also be aided by the presence or absence of specific skeletal traits, such as the development of pubic symphysis or the existence of a prominent Adam's apple in males. It is important to remember that even though these skeletal indicators offer insightful data, individual variance and population-specific variations may affect them [12]. Skeletal morphology can be influenced by a variety of circumstances, including age, health, and lifestyle choices, which may make gender assessment more difficult. Thus, in order to obtain the most accurate gender estimation from human skeletal remains, forensic anthropologists must use a comprehensive strategy that takes into account a variety of skeletal characteristics and circumstances [13].

An important component of bioarchaeology and forensic anthropology is gender estimation, which helps identify people who are unknown in archaeological or forensic contexts. The gonial and antegonial angles are two anatomical traits that have received a lot of attention among those used to estimate gender since they might be seen as good predictors of sexual dimorphism [14]. The purpose of this study is to examine the use of gonial and antegonial angles in gender assessment, as well as their significance, methodology, limitations, and ramifications. The angle created by the mandibular body and mandibular ramus intersecting is known as the gonial angle [15]. Drawing lines between the anterior and posterior ends of the ramus and the bottom border of the mandible is how it is measured. On the other hand, the inferior border of the mandible and the antegonial notch cross to produce the antegonial angle. Differences in mandibular size and shape, which are linked to sexual dimorphism, have an impact on both angles [16].

The morphological variation between males and females of the same species is known as sexual dimorphism. Skeletal sexual dimorphism in humans is characterized by differences in bone structure, especially in the pelvis and head. Although gender can be inferred from the pelvis, the mandible can also be used as a source of information, particularly in situations where the pelvis is insufficient or unavailable [17]. Males and females differ in the gonial and antegonial angles, a feature of sexual dimorphism that may be measured via morphometric analysis. Anthropometric methods, including both manual and digital ones, are usually used to measure gonial and antegonial angles. When taking manual measurements, the angles on skeletal remains are measured directly using calipers or protractors [18]. Utilizing imaging technologies like computed tomography (CT), radiography, or three-dimensional (3D) scanning are examples of digital approaches. Software tools make it easier to measure accurately and allow comparisons with databases of people who are known to be of a certain sex [19].

Despite their potential benefits, gonial and antegonial angles present a number of drawbacks and difficulties when it comes to gender estimation. They must be acknowledged, though. Although these angles show substantial sexual dimorphism in the population, interpretation can become more difficult due to individual variance, age-related changes, and environmental influences. The use of skeletal remains to estimate gender calls for a rigorous assessment of sample representativeness and the possibility of bias in forensic casework [20]. Variations in measurement technique, individual variances in anatomy, and variations in observer interpretation can all lead to variability in angle measurements. Mandibular morphology can be influenced by age, demographic ancestry, and nutritional state, which could make sex prediction based only on angle measures more difficult. To guarantee the accuracy and application of sex estimation techniques based on gonial and antegonial angles, reference datasets with a variety of population compositions must be made available [21].

Methodological considerations may have an impact on the precision of gender estimation utilizing gonial and antegonial angles. The reliability of results can be impacted by inconsistencies introduced by variations in measuring methodologies, such as disparities in landmark recognition or angle-calculating methods. Strict quality control procedures and standardization of measurement techniques are necessary to reduce errors and improve sex estimation accuracy. There can be considerable overlap between males and females, especially in diverse or mixed populations, even if gonial and antegonial angles may show sexual dimorphism at the population level. Gender assessment utilizing mandibular angles can be more accurate and reliable with the use of imaging technology advancements like geometric morphometrics and 3D scanning. Statistical modeling methods and machine learning algorithms can be integrated to improve the accuracy of angle measurements and streamline automated sex estimation procedures. Moreover, multidisciplinary partnerships involving forensic anthropologists, radiologists, anatomists, and computer scientists are necessary to improve current practices, set uniform guidelines, and develop reference materials that cover a range of global populations [22].

Conditions like postmortem changes and mandibular damage may restrict the usefulness of gonial and antegonial angles for estimating gender. The accuracy of angle measurements might be hampered by postmortem alterations such as bone loss, erosion, and fragmentation, which can also obscure anatomical landmarks. Similar to this, severe injuries to the mandible, like fractures or dislocations, can change how it is shaped and how angles are understood. In forensic and archeological contexts, the gonial and antegonial angles are useful anatomical traits for estimating gender. These angles provide a non-destructive and accessible way to evaluate sexual dimorphism in skeletal remains, notwithstanding their inherent limitations and difficulties [23]. For skeletal sex estimation to progress and become more useful for forensics, studies on population-specific variation, measurement technique refinement, and integration of sophisticated imaging and computational tools are necessary. Ingaleshwar et al. stated that there were statistically significant mean differences between the two sides (left and right) in males and females with respect to gonial angle and also condyle-coronoid angle when measured on both right and left sides in males and females, showing significant mean differences with p<0.05. The gonial angle was found to be more statistically significant in females in comparison to males, and similarly, the condyle-coronoid angle was found to be higher in females than males, which shows it can be used as a significant parameter in determining gender [24]. The present study showed the mean comparison values between males and females based on the gonial angle and antegonial angle (in degrees). Gonial angle statistical analysis revealed no significant difference between males and females (p=0.679). Both males and females showed statistical significance in the antegonial angle, with p-values of 0.008 and 0.001, respectively.

Limitations

Gonial and antegonial angles have limits, even if they have the potential to be useful additional markers for estimating gender. When evaluating these angles, forensic anthropologists and orthodontists should proceed with caution and take into account the context of a thorough analysis that includes demographic data and a variety of skeletal indications. To improve the accuracy and reliability of gender estimation using gonial and antegonial angles, more research is required to standardize measuring methods and validate them across a range of populations.

## Conclusions

Sex identification from accessible skeletal remains has important implications for both medicolegal and anthropological research. The current study used parametric analysis, namely the gonial and antegonial angles, to determine the gender of the South Indian population. These metrical criteria, applied in conjunction with morphological characteristics, may prove to be a helpful tool for mandibles. Further research involving different demographic groups may be beneficial in identifying racial and ethnic distinctions through the use of the mandible. Larger sample sizes in studies could be useful in correlating gender determination among South Indians utilizing metric factors or morphology.
